# Choices and Challenges: Visualizing Contraceptive Use Dynamics Data in 15 Low- and Middle-Income Countries

**DOI:** 10.9745/GHSP-D-22-00212

**Published:** 2023-06-21

**Authors:** Eve Brecker, Dana Sarnak, Kaitlyn Patierno

**Affiliations:** a Population Reference Bureau, Washington, DC, USA.; b Johns Hopkins Bloomberg School of Public Health, Baltimore, MD, USA.

## Abstract

Interactive data visualization tools, particularly Sankey diagrams, are an effective approach for showing high-level trends in contraceptive adoption, switching, and discontinuation.

## Key Messages

- Data on contraceptive use dynamics are difficult to measure, analyze, and present, especially to policy audiences.
- Visualization tools, such as Sankey diagrams, are one way to better connect policy audiences with easily understandable contraceptive use dynamics data that will support them in centering women’s needs and preferences during program design and implementation.
- Users found the Choices and Challenges tool to be an alternative approach for introducing the complex topic of contraceptive adoption, discontinuation, and switching to policy and program decision-makers.
- The Choices and Challenges tool has been effectively used to ensure contraceptive discontinuation and switching are key considerations within national policy documents.
- The Choices and Challenges tool can be an effective, evidence-based resource that enables policy and program decision-makers to deliver high-quality, client-centered care that supports contraceptive autonomy and choice.

## INTRODUCTION

Family planning (FP) programs are high-impact interventions that can improve the health and well-being of women worldwide. To align with global commitments and strategies, FP programs often intrinsically emphasize initiating new contraceptive users. Yet, over the course of their lives, women will choose to start, stop, or switch contraceptive methods to meet their reproductive needs and preferences. Therefore, it is important to understand the dynamics of contraceptive use—including method adoption, switching, and discontinuation—to effectively support women and couples in achieving their FP goals. For example, studies indicate that, on average, more than one-third of women who start using a modern contraceptive method stop using it within the first year, and more than one-half stop before 2 years.^1^ Such discontinuation can be an important determinant of modern contraceptive prevalence rates and outcomes like unintended pregnancy. Expanding the focus of FP programs to fully address existing users and their contraceptive decisions will strengthen the quality and reach of FP service delivery investments.

Data on contraceptive use dynamics are often poorly understood and can be challenging to use without advanced demographic training. Moreover, robust data on contraceptive use dynamics are limited in many contexts. One of the few comprehensive sources of these data exists within the Demographic and Health Survey (DHS) reproductive and contraceptive calendar. For many countries, this calendar provides the most detailed evidence on women’s reproductive behavior and key reproductive events for a multiyear period. It is a critical resource for policy and program decision-makers (hereafter referred to as “decision-makers”) seeking to understand contraceptive use dynamics. However, analysis of contraceptive use dynamics—for example, discontinuation rates or examination of reproductive outcomes after discontinuation—are most often presented in research reports and peer-reviewed journal articles, which are generally intended for academic audiences and less frequently framed with decision-makers in mind. Therefore, in this article, we describe the development of a novel data visualization resource on contraceptive use dynamics in 15 low- and middle-income countries (LMICs) and share ways the tool can be leveraged for program- and policy-related activities.

We describe the development of a novel data visualization resource on contraceptive use dynamics in 15 low- and middle-income countries and share ways the tool can be leveraged for program- and policy-related activities.

## DEVELOPMENT OF A DATA VISUALIZATION RESOURCE ON CONTRACEPTIVE USE DYNAMICS

We sought to improve the global knowledge base on contraceptive use dynamics in 15 LMICs with recent DHS data by using creative data visualization approaches that depicted the complex dynamic patterns of contraceptive use. In the exploratory research for designing our resource, we knew of only 2 other tools in development that used DHS contraceptive calendar data to visualize patterns of contraceptive continuation, switching, and discontinuation. The first tool uses a chord diagram to demonstrate the transition, or “churn,” of new users from contraceptive use to nonuse and between types of method used, as well as their reasons for discontinuation.^2^ The second tool, developed by iSquared, uses the Sankey diagram in an interactive web platform to show 12-month continuation rates by method and discontinuation rates divided into “in need” and “not in need.”^3^ One limitation of these tools is the specificity in their methodological samples and approach. The chord diagram sample is limited to new users and excludes nonusers or women who discontinue to get pregnant. The iSquared tool uses an episode-based analysis, which can be difficult to interpret without demographic training. Further, both tools use data from the full 5-year contraceptive calendar for which there is evidence of decay in quality due to recall bias.^4^

Because of these limitations, after consultation with leading experts on contraceptive use dynamics, we aimed to create a data visualization resource, the Choices and Challenges tool, that built upon both existing tools in several ways. First, we chose the Sankey diagram as the main component of the visualization to illustrate contraceptive use dynamics due to its simplicity and ability to show 2 time transitions. The Sankey diagram is a type of flow diagram that shows directional transitions and relative size of flows between specific categories or “nodes.” Second, we opted to use the woman as the unit of analysis for ease in interpreting the data for program implementers and policymakers. Third, we included a feature to stratify the Sankey diagram by age group because contraceptive use varies significantly over the life course. Additionally, we included a horizontal bar chart to illustrate the reasons for discontinuation and the wantedness status of pregnancies within 12 months of discontinuation while still in need. Lastly, we included women who were not currently using a method at the start of the calendar to analyze uptake and continuation, or lack thereof, over the 2-year period.

The data shown in this visualization tool are derived from the DHS—nationally representative household surveys conducted approximately every 5 years—which collects information on key population and health indicators.^5^ Specifically, the tool we developed used data from the DHS contraceptive calendar module, recording women’s reproductive status in each month covering the 5-year period before the survey. Reproductive status options include pregnancy, birth, termination, and contraceptive use or nonuse. In any month when a woman reported discontinuing a contraceptive method, she was asked why she discontinued, and her reason was recorded. We included data from 15 LMICs: Bangladesh, Benin, Burundi, Ethiopia, Guinea, Kenya, Liberia, Malawi, Mali, Myanmar, Nigeria, Rwanda, Senegal, Uganda, and Zambia.

To examine adoption, discontinuation, and switching patterns, we extracted data from each country’s latest DHS using the most recent 2-year period (months 3–27) of the full 5-year contraceptive calendar. This time period was chosen to analyze the most data available without sacrificing data quality, based on findings from calendar quality assessments performed by Bradley et al. that revealed a decay in calendar quality due to recall bias over longer time periods.^4^ We excluded the most recent 3 months of calendar data to account for the possibility that a woman was pregnant and did not yet know it, as is standard in DHS analysis.^1^ Finally, we excluded women who reported using male or female sterilization.

### Choices and Challenges Tool

Using the Sankey diagram enabled us to demonstrate the changing nature of women’s FP decisions across different locations, times, samples, and methods, similar to Choi.^3^ Data on contraceptive discontinuation and switching are often reported as discontinuation rates, specifically, 12-month discontinuation rates.^6^^,^^7^ These rates are generated from hazard models using “episodes of use” (the duration in months of continuous use of the same methods) as the unit of analysis. However, in line with recent research that promotes studying contraceptive discontinuation at the woman- rather than episode-level, we opted to present contraceptive use dynamics—including adoption, discontinuation, and switching—using individual women as the unit of measurement in the Sankey diagrams.^8^ We built transition matrices by extracting the reproductive status of each woman in the sample at 27 months, 15 months, and 3 months before the survey using her contraceptive calendar. Our analysis accounts for the sampling design (stratified 2-stage cluster) of the survey and uses the sample weights provided by the DHS to generate a weighted sample size (“N”) for patterns of contraceptive use, at each of the 3 time intervals, with 12 months between each interval. We do not provide confidence intervals for the transition matrices, given that Sankey diagrams are not meant to be used for statistical inference.

The Choices and Challenges Tool shows contraceptive use dynamics using individual women as the unit of measurement in the Sankey diagrams.

The Choices and Challenges tool presents data for 3 samples of women, defined by their status at the start of the abbreviated 2-year calendar: (1) all women, (2) current FP users, and (3) non-FP users. Because contraceptive use varies significantly over the life course, we also stratify the Sankey diagrams by age group for women aged 15–24 years, 25–34 years, and 35 years and older.

For the sample that followed current FP users, we also present the percentage of women who discontinued while still in need during the 2-year observation period. We classified a woman’s reason for discontinuation as in need or no longer in need according to Staveteig et al.^9^ We define in need as discontinuation due to: method failure (e.g., becoming pregnant while using); method-related reasons, cost/access, opposition, or other reasons unrelated to lack of need.^9^ We define no longer in need as discontinuation due to the desire to become pregnant, marital dissolution, infrequent sex/husband away, and menopause.^9^ Of note, although the definition of in need and no longer in need is somewhat standardized in the FP community, the DHS does not have data on a woman’s fertility preferences at the time of discontinuation. Therefore, we acknowledge that classifying women as in need is an assumption with limitations.

### Contraceptive Use Dynamics: Adoption, Switching, and Discontinuation

**FIGURE 1:**
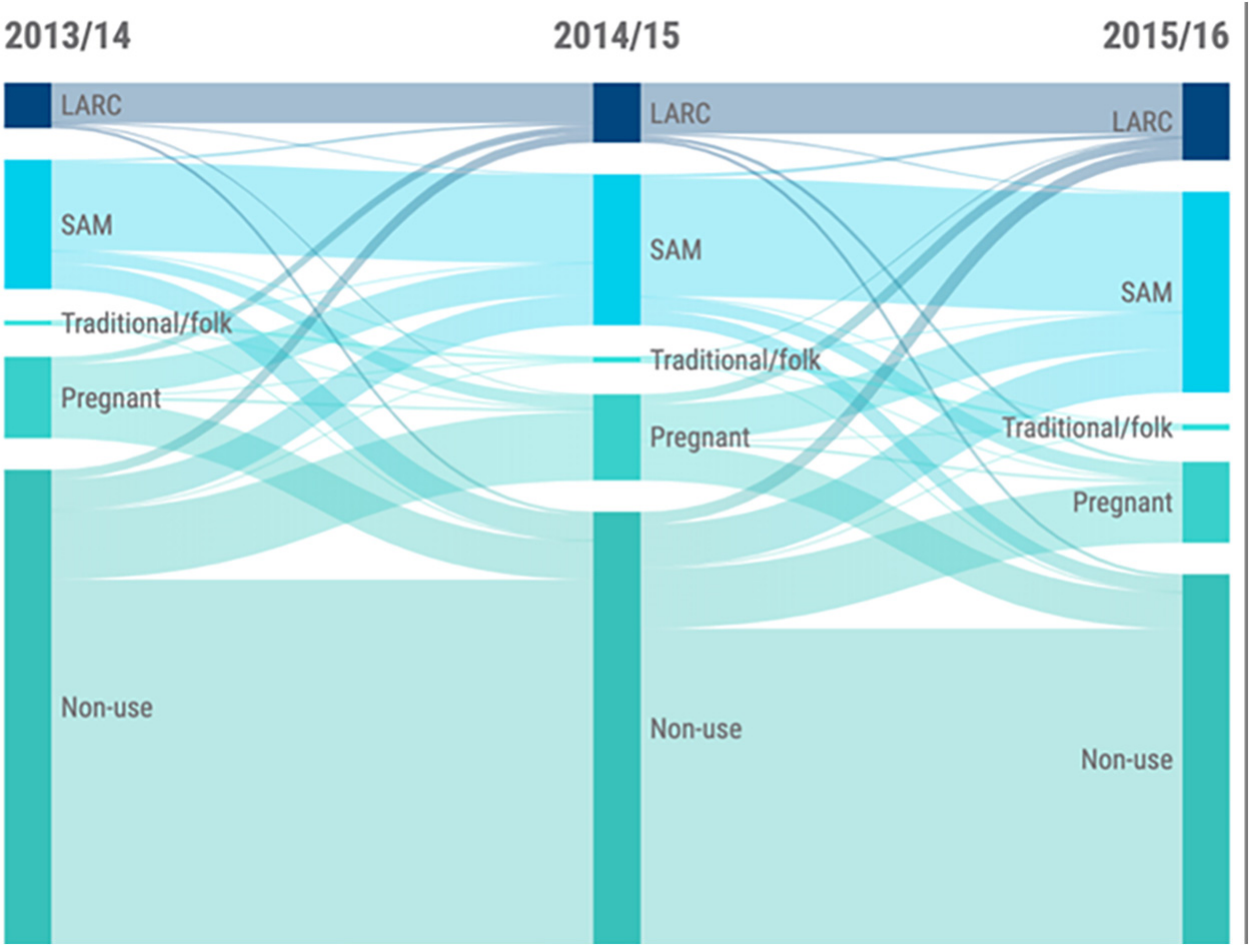
Sankey Diagram Showing Family Planning Use Status for Women Aged 15–49 Years in Malawi, From 2013/2014 to 2015/2016

To better understand how visualizing these data within the national context may allow policy and program audiences to make informed decisions, we will take a deeper look at the visualization and key trends identified in the analysis for Malawi. When examining all women ages 15–49 years in Malawi between 2013/2014 and 2015/2016, FP use (excluding sterilization) in Malawi increased from 24% to more than 38% (Figure 1). An estimated 20% of long-acting reversible contraception (LARC) users and 33% of short-acting modern method users were no longer using FP or were pregnant in 2015/2016. Among the 7% of women who switched methods by 2015/2016, the majority (68%) transitioned to a more effective method.

**FIGURE 2:**
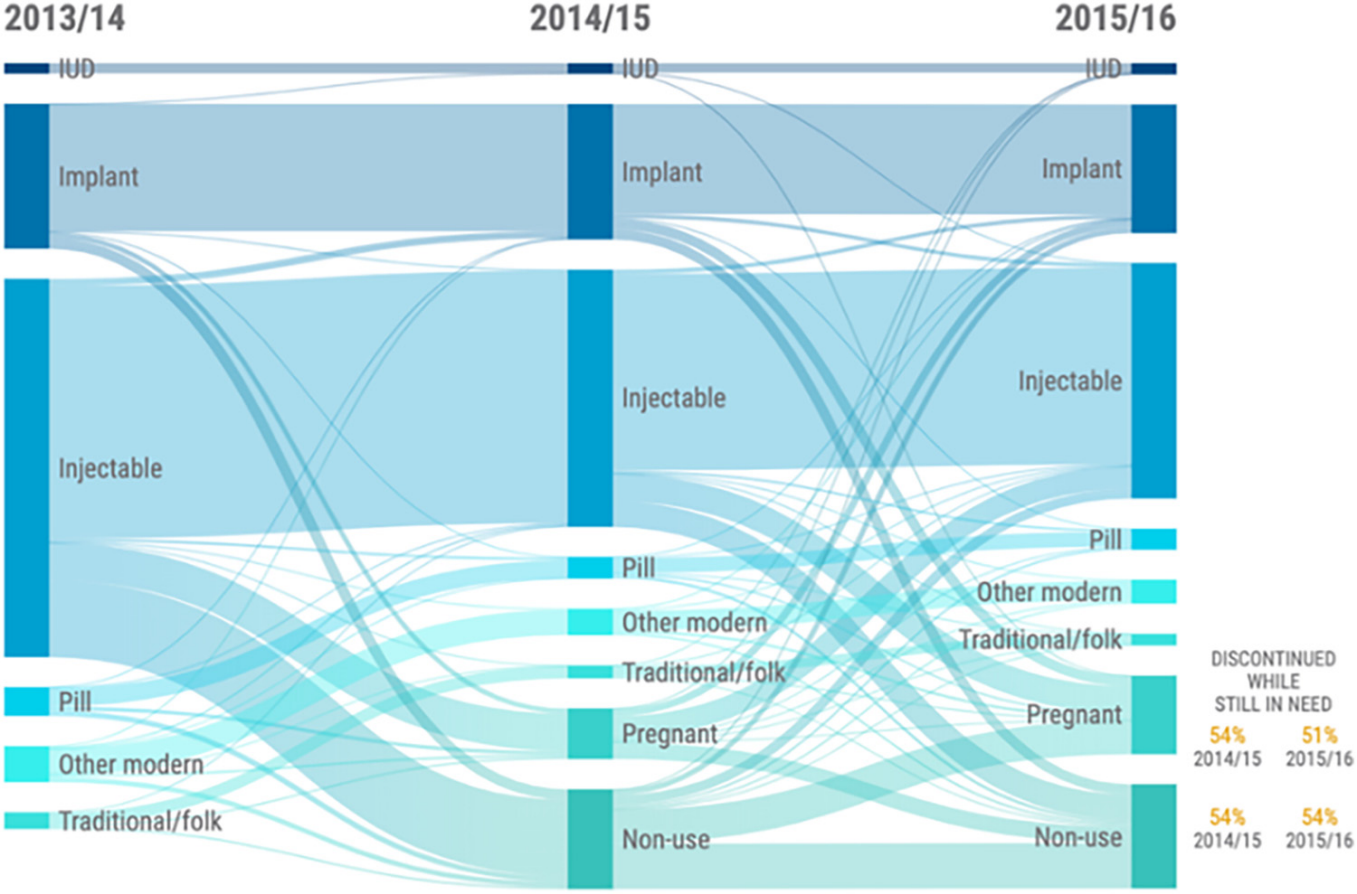
Sankey Diagram Showing Family Planning Use by Method Type for Women of All Ages in Malawi, From 2013/2014 to 2015/2016

As shown in Figure 2, nearly 30% of women who were using FP in 2013/2014 in Malawi were no longer using any method or were pregnant in 2015/2016. Among those no longer using FP in 2015/2016, 51% of pregnant women and 54% of nonusers reported discontinuing their most recent method while still in need. Of the women using an intrauterine device or implant in 2015/2016, 19% switched from other methods.

**FIGURE 3:**
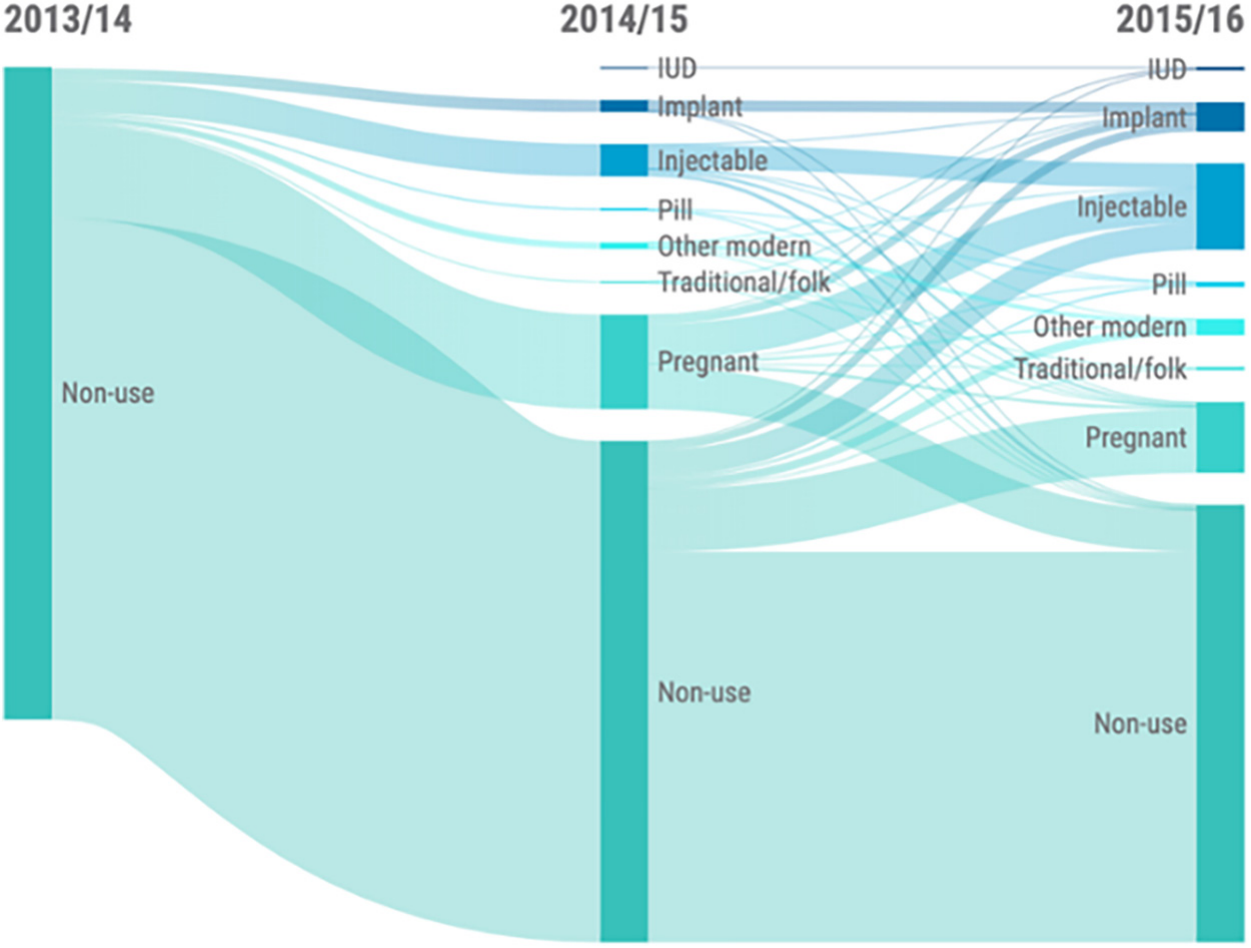
Sankey Diagram Showing Women of All Ages Not Using Family Planning in Malawi, From 2013/2014 to 2015/2016

An estimated 78% of women using no method of FP in 2013/2014 in Malawi remained nonusers or became pregnant in 2015/2016 (Figure 3). An estimated 17% initiated use of short-acting modern methods, while 5% initiated use of LARCs. Among women who began using FP between 2013/2014 and 2015/2016, injectable contraceptives were the most popular method across all age groups.

### Reasons for Discontinuation

Although Sankey diagrams were selected as the most interpretable visualization approach for measuring individual women’s contraceptive status change, this approach also created limitations in how to present data on reasons for discontinuation. Specifically, the multiple cross-sections that showed women’s status at 3 time periods meant that each reason could not be linked consistently to discontinuations captured in the Sankey diagrams. Because reasons for discontinuation could not be connected to the transitions in the Sankey diagrams, we elected to show the distribution of reasons for discontinuations using the standard episode-based approach using the 2-year calendar data in a horizontal bar chart. This approach further helped to mitigate sample size limitations when exploring reasons for discontinuation in disaggregated groups.

**FIGURE 4:**
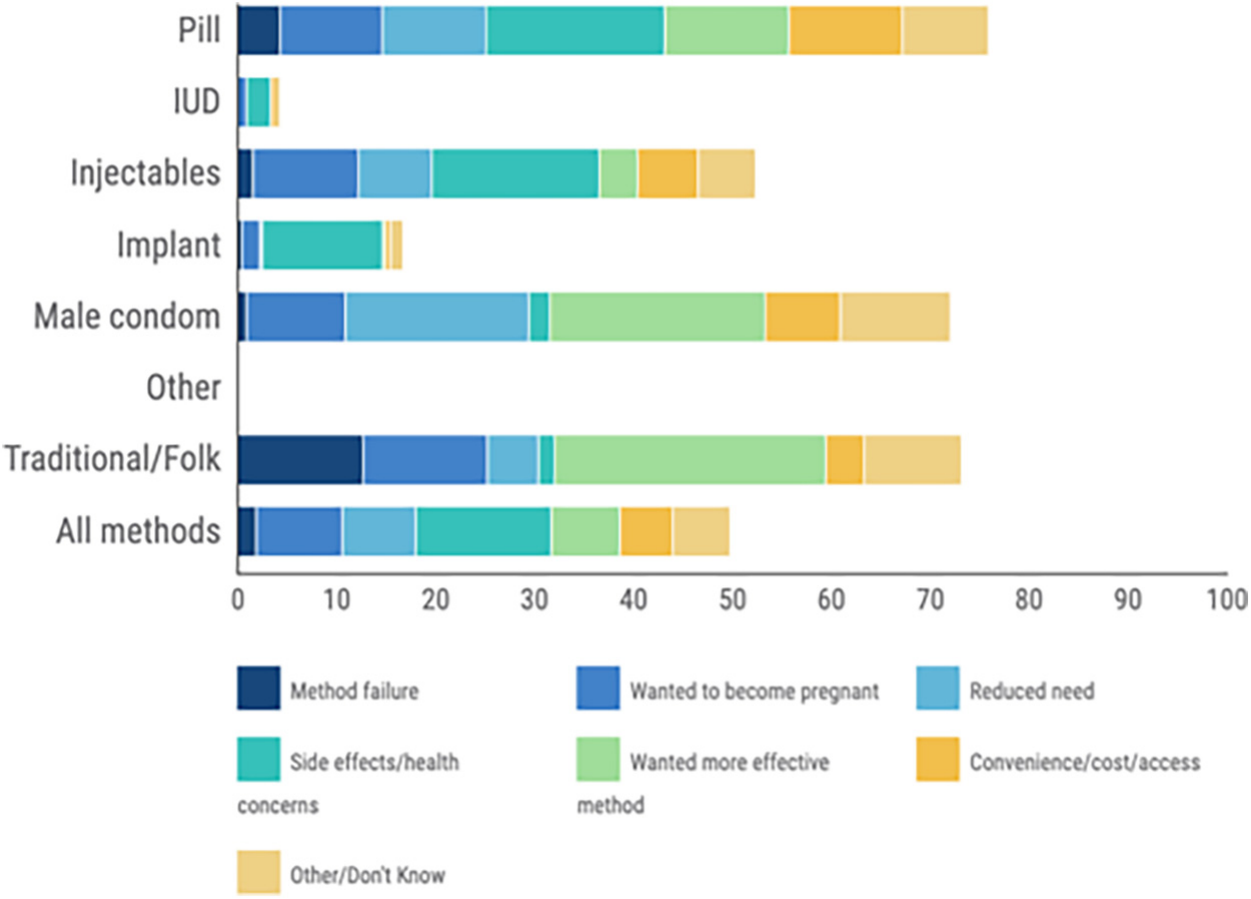
Reasons Reported by Women of All Ages for Discontinuing Family Planning During the Second Year of Use in Malawi^*a*^ ^*a*^Analysis based on episodes of contraceptive use. Methods based on fewer than 100 episodes are not shown.

Referencing our Malawi example, women using the pill or injectable were most likely to discontinue due to side effects or health concerns (Figure 4). However, women using male condoms or traditional methods were most likely to discontinue because they wanted a more effective method.

### Wantedness of Pregnancy After Recent Discontinuation While Still in Need

Pregnancy intention (defined as “wanted then,” “wanted later,” or “not wanted at all”) related to discontinuation while still in need was analyzed using the latest DHS individual and birth recode files for each country to create a sample of women who discontinued while still in need (1) in the 3 years before the survey and (2) who became pregnant within 12 months of the respective discontinuation.^10^ We then looked at the intention status of the subsequent pregnancy retrospectively reported by the woman at the time of the survey. The analysis is limited to pregnancies that resulted in live births.

**FIGURE 5:**
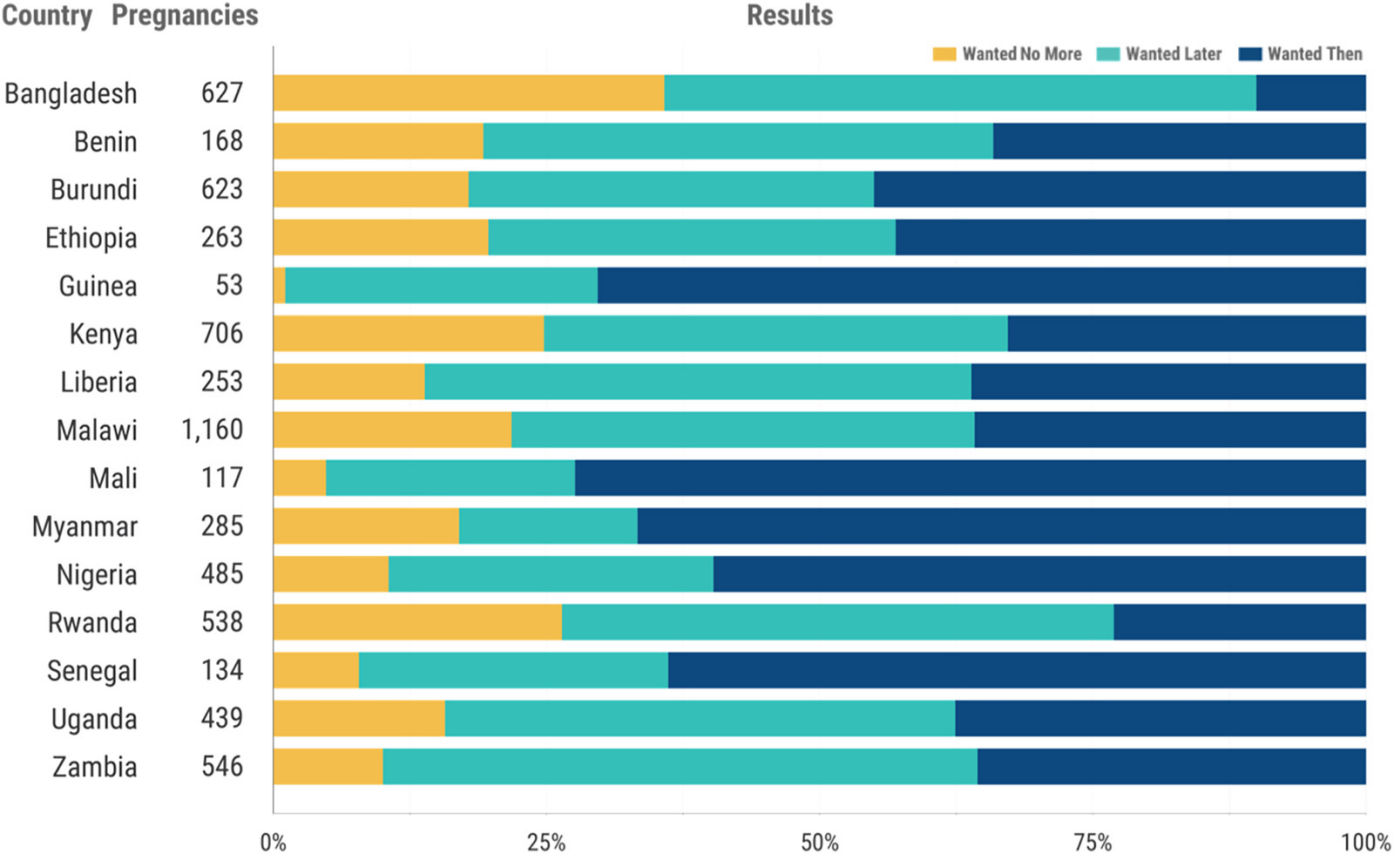
Wantedness of Pregnancies Within 12 Month of Discontinuation While Still in Need

In some countries, more than 60% of pregnancies that begin within 12 months of discontinuation while still in need are unwanted or mistimed. In Malawi, fewer than 40% of pregnancies that occur within 12 months of discontinuation while still in need were reported to be intended at the time (Figure 5).

### Policy Recommendations

To help make these data actionable, we conducted a literature review to identify policy and program interventions associated with enhancing contraceptive continuation in LMICs for women wanting to avoid pregnancy. We identified a total of 20 records from database searches that met our inclusion criteria. Of these, 15 sources supported improved counseling,^11^^–^^25^ 8 sources reported improved access to methods, ^11^^,^^21^^,^^23^^,^^26^^–^^30^ and 7 sources reported better utilization of follow-up mechanisms.^11^^,^^12^^,^^17^^,^^22^^,^^23^^,^^25^^,^^30^ We also noted recurring evidence of encouraging more male involvement in decision-making,^16^^,^^21^^,^^25^^,^^29^^,^^30^ using job aids as a technique to improve counseling,^14^^,^^17^ promoting self-care as a technique to improve access,^21^^,^^27^^,^^29^^,^^30^ and addressing cost as a barrier to access^11^^,^^19^^,^^23^^,^^26^^,^^28^ —all with less evidence but still notable in our findings. These policy recommendations are found at the bottom of the tool in a collapsible list that promotes policy action on country-specific trends observed within the tool. These recommendations are intended to support decision-makers in designing and implementing interventions that center the client’s stated reproductive health needs and preferences.

Considering the trends that we observed in Malawi’s data (i.e., approximately 50% discontinuing while still in need; approximately 20% of LARC users switching from another method; and short-acting method users citing side effects as the reason for discontinuation), when presenting to policy audiences, we might offer the recommendation concerning improved contraceptive counseling as an approach to ensure clients are fully informed on all of their method options and the side effects associated with each option. We might also offer the recommendation of improved follow-up mechanisms so that if a client is experiencing unwanted side effects, she has an opportunity to discuss other options (especially those that are more effective, if that is a concern) with a provider should she want to continue avoiding pregnancy. Of course, there are other recommendations that could also be implemented in place of or in tandem with improved contraceptive counseling and improved follow-up mechanisms. Decision-makers should complement these data trends with their country’s context and other data sources available to them to ensure they advocate for the most effective program or policy changes for their settings.

### Maintenance

Initially, we launched the tool with 9 donor-prioritized countries. Population Reference Bureau (PRB) manages the tool and updates countries when new DHS are released. PRB adds countries based on the availability of new data that include the contraceptive calendar and prioritizes countries with discontinuation rates of more than 20%. Since 2019, we have added 6 countries and updated data for 3 countries.

## PRACTICAL APPLICATION AND USE OF THE CHOICES AND CHALLENGES TOOL

Enhancing contraceptive continuation for women wanting to avoid pregnancy within the dynamic context of women’s reproductive lives by clarifying the barriers, challenges, options, and solutions helps decision-makers better meet women’s FP needs. The visualization resource is intended to be a starting point for policy and program discussion and the basis of further study rather than a comprehensive analysis. Since its launch, the tool has been presented at 3 national policy convenings in Senegal, Kenya, and Nigeria, and a technical workshop for alumni of the Policy Communications Fellowship program of the Policy, Advocacy and Communication Enhanced for Population and Reproductive Health’s, also known as the PACE Project.

The tool is intended to be a starting point for policy and program discussion and the basis of further study rather than a comprehensive analysis.

In August 2020, PRB presented the tool to a group of 10 government officials in Senegal that included both the Ministry of Health’s Director of FP Division and the National Agency of Statistics and Demography. In July 2021, on the invitation of the Director of the National Council for Population and Development, PRB presented the tool to a virtual group of more than 80 participants in Kenya during a quarterly meeting of the FP Advocacy Technical Working Group, which included key participants from the global and national public and private health sectors. Finally, the tool was presented in Nigeria in September 2021, at the invitation of the Ministry of Health Director of Reproductive Health, at a quarterly meeting of the Reproductive Health Technical Working Group. The audience was composed of more than 40 government/private public health stakeholders. After the presentations, each of the 3 groups was guided through a discussion of the implications of the tool’s national findings to the ongoing work around contraceptive uptake and discontinuation within the countries to tailor recommendations for potentially enhancing contraceptive continuation.

PRB also hosted a training of trainers workshop for 17 alumni of the PACE Project’s Policy Communications Fellowship program on the tool’s utility. The workshop intended to provide implementers—professionals who interact with relevant decision-makers—with a complementary resource to build compelling cases for enhancing contraceptive continuation for women who want to avoid pregnancy. After a presentation on the tool, participants engaged in 2 exercises that allowed them to explore how they might use the tool in their current work. Participants provided feedback on their user experience through a real-time debrief discussion (where the facilitators took notes on salient points) and an online post-workshop survey (Supplement).

Through both types of sensitization events (national presentations and trainings), PRB has been able to present visualizations of data in a way that participants found useful in informing their subsequent programmatic decision-making process. This usefulness has been demonstrated at the national level in 2 instances.

In Kenya, PRB’s presentation to the quarterly technical working group elicited an invitation on behalf of the Director of the National Council for Population and Development to provide technical input on Kenya’s 2021–2024 FP Costed Implementation Plan (CIP). The technical working group reported that the tool presented a compelling argument for considering contraceptive discontinuation and switching. Further, PRB received feedback during the meeting that the tool underscored the importance of better considering the needs of current FP users and addressing obstacles to contraceptive continuation for women wanting to avoid pregnancy in Kenya’s FP strategy. PRB’s lead technical working group presenter, who works closely with the Ministry of Health, notified PRB that the presentation directly contributed to the programmatic considerations of contraceptive discontinuation in the FP CIP. Although the FP CIP has yet to be publicly released at the time of writing, a published dissemination brief includes a discontinuation-focused key performance indicator (6.1.1.a) in its results framework.^31^

In Nigeria, the Director of Reproductive Health affirmed that the data PRB presented validates Nigeria’s FP2030 recommitment and should inform implementing organizations’ strategic plans. Participants were interested in linking the tool data to a Bill & Melinda Gates Foundation study that found that the greatest barrier to continuation was myths/misconceptions related to side effects, and they were interested in linking the data to stock-outs as a reason for discontinuation.^32^ Although these are 2 linkages that we cannot specifically draw from the DHS data, generating interest in the reasons for discontinuation through the visualization may encourage increased exploration of such policy-relevant research topics.

After the training of trainers workshop, PRB provided seed funding through small grants to support participants in using the tool to achieve their advocacy goals. The following examples are ways in which the tool is being applied.

- An August 2022 policy brief on FP use and discontinuation among women aged 15–24 years in Kenya.^33^
- This brief has been disseminated to about 3,500 people across Kenya's National Council on Population and Development, as well as a variety of Ministry of Health technical work groups, to include: National Reproductive Health; Maternal Health; and National and County End Teenage Pregnancy.
- A cartoon video explaining trends in discontinuation for women younger than 25 years in Nigeria and subsequent policy recommendations.
- A policy brief examining the ability of pharmacy providers in Nigeria’s Ekiti State to provide high-quality counseling on available contraceptive methods.
- Two video shorts illustrating (1) the dynamic nature of contraceptive behavior among Nigerian women (younger than 25 years, 25–34 years, and 35 years and older) through a short tutorial on the tool and (2) why Nigeria’s FP counseling manual should incorporate a life-stage focus by 2023. These videos, created by Funmi OlaOlorun from the University of Ibadan in Nigeria, are available on PRB's website (https://www.prb.org/resources/contraceptive-use-dynamics/).
- An evidence-based slide deck demonstrating why the health sector should prioritize contraceptive use among young women in Malawi

Overall, reported feedback on the tool after the presentation included that it was easy to learn and understand, findings validated existing national research and therefore provided additional support for decision-making, and that the ability to replicate or customize the tool to national needs was critical (through supplemental PRB technical assistance). All 3 national audiences lauded its utility in helping participants to understand major national discontinuation and switching trends and the extent to which discontinuation affects method choice, uptake, and annual changes in modern contraceptive prevalence rates. Drawbacks included that there was sometimes not as much nuance in the findings for decision-makers to fully understand use trends, the tool could not further disaggregate age groups beyond those that were already featured, and the tool could not provide information on sociodemographic characteristics of the samples. Both decision-makers and implementers found the tool easy to use and understand, refreshing in its innovative format, and an improvement in simplifying complex data compared to more traditional approaches that use complicated indicators and number-heavy graphics.

## DISCUSSION

The Choices and Challenges tool provides an alternative visualization approach for decision-makers, researchers, program implementers, and FP advocates to review powerful evidence behind national trends in contraceptive use. Analyzing data at the person level rather than using standard discontinuation rates and shortening the duration of data used to 2 years were both strategies that effectively made contraceptive use dynamics trends more easily understood by nonscientific audiences and methodologically stronger, respectively.

Users were enthusiastic about how our tool’s Sankey diagrams underscored the need to look beyond contraceptive prevalence to understand the dynamic patterns of women’s contraceptive use. These visualizations highlight the need for programs that are more responsive to women’s changing contraceptive needs and preferences. Historically, FP policies and programs inherently tend to prioritize new users. Our tool’s Sankey diagrams offer a more comprehensive visualization of FP users and non-users, drawing attention to the need for programs that are more responsive to current users and new users. The tool offers a resource for advocates to make the case that FP policies and programs need to better incorporate considerations about current users, including drawing upon the interventions listed in the recommendation section. Once users were provided a brief overview and tutorial on how to read the tool, they could apply it and use the analysis for decision-making or for communicating critical data trends for decision-making.

Users were enthusiastic about how the Sankey diagrams underscored the need to look beyond contraceptive prevalence to understand the dynamic patterns of women’s contraceptive use.

Since the launch of our tool, we noted that the Sankey diagram had been used to show contraceptive use dynamics using panel survey data. The Sankey diagram was used in a static form to show “change in contraceptive use” between broad categories of pregnant women not using FP and using FP samples over 1 year and changes in broad method categories (e.g., no use, traditional, short acting, and long acting) over 1 year.^34^ Since this brief used Performance Monitoring for Action data rather than DHS data, the data did not suffer from recall bias limitation as the Choices and Challenges tool does using retrospective calendar data. Although in this application, the diagrams effectively show transitions in women’s contraceptive use, they illustrated a limited period of time and in less depth than our approach. Here, we were able to expand our visualization tool to effectively represent the flow of women across time.

The Choices and Challenges tool can complement the use of other FP program and policy decision-making tools, such as Avenir Health’s Impact Matrix and FP Goals model, by providing alternative visualizations on contraceptive use dynamics to inform prioritization of investments. Where discontinuation rates are high and contribute significantly to unintended pregnancy, decision-makers should ensure discontinuation is addressed in policy and program guidelines and can refer to evidence-based interventions, such as those highlighted in the Choices and Challenges tool, and further elaborated through tools such as the FP Goals model.^35^ The Choices and Challenges tool offers a full-service package for introducing national contraceptive use dynamics evidence for decision-making in that it shows quantitative data complemented with qualitative recommendations for action. Accompanied by downloadable graphics and available STATA.do files for calendar quality assessments, select components of the tool were intended to be replicated in any way that meets local stakeholders’ needs. At the time of writing this article, the tool had been disseminated across 4 countries supporting 1 national policy change and across 17 Anglophone Africa practitioners with plans to innovate and use the tool in 3 additional countries.

### Limitations

Three of the main limitations of this tool relate to the quality of calendar data and retrospective reporting of pregnancy wantedness. First, retrospective calendar data generally underestimate modern contraceptive prevalence rates due to recall bias; it will rarely exactly match current use status of cross-sectional DHS data. Similarly, the visualization will most likely represent an underestimate of all use, switching, and discontinuation. Second, reasons for discontinuation, on which we base categorization of in need versus no longer in need, are limited in that women are only allowed to report 1 reason for discontinuation, and fertility preferences at the time of discontinuation are not explicitly recorded. Therefore, our classification of a woman being in need or no longer in need at the time of discontinuation is an assumption. Finally, another limitation of the tool is its use of retrospective reporting on pregnancy wantedness status. The DHS asks women whether the wantedness status of a current pregnancy or live birth that occurred within the calendar period was “wanted then” (intended), “wanted later” (mistimed), or “wanted no more” (unintended). Recording pregnancy wantedness, particularly post-birth, may not be reflective of a women’s intentions at the time she became pregnant. There is evidence that prospective versus retrospective reporting of pregnancy wantedness can differ; the latter can be subject to post-rationalization bias.^36^^–^^38^

It is important to note that analyses of this type of data are extremely nuanced by nature. As such, there are a plethora of ways that we could have more specifically explored how to make our work more detailed, especially in the Sankey diagrams, such as using shorter intervals/more points in time or showing the individual trajectories of women. However, a primary objective of our tool was to balance detail with the tolerance of our key audience: policy and program decision-makers. As such, and with a limited budget, more complex analyses—using multistate life tables, latent transition matrices, or sequence analysis—were considered but determined to be beyond the scope of this tool, audience, and project funding.

## CONCLUSION

By understanding the dynamics of contraceptive use, including why women who wish to avoid or delay pregnancy discontinue, decision-makers can better deliver high-quality, client-centered services that enable women and couples to make the best FP choices for themselves. Applying analytical approaches that simplify the interpretability of results along with straightforward visual tools to highlight key trends may increase the use of data on contraceptive use dynamics, including DHS calendar data, by decision-makers. Sankey diagrams are one such approach to demonstrate women’s complex, continuously changing patterns of contraceptive trajectories over time and to provide insights into major trends in contraceptive discontinuation and switching that can—and should—influence FP policy and program decisions.

Studies regarding the specific impact of policy approaches on contraceptive use dynamics are limited. However, findings drawn from the programmatic literature point to key policy and program considerations. Studies suggest that investments to improve comprehensive counseling, reduce financial and other barriers to access, and expand the FP method mix may help enhance contraceptive continuation for women who want to avoid pregnancy and reduce unintended pregnancy.

## Notes

**Peer Reviewed**

**First published online:** June 20, 2023.
